# Reports from the front lines: *Field Notes*, a new JIAS feature

**DOI:** 10.1002/jia2.25966

**Published:** 2022-07-25

**Authors:** Kenneth H. Mayer, Annette H. Sohn, Marlène Bras

**Affiliations:** ^1^ The Fenway Institute and Harvard Medical School Boston Massachusetts USA; ^2^ TREAT Asia, amfAR – Foundation for AIDS Research Bangkok Thailand; ^3^ Journal of the International AIDS Society Geneva Switzerland

Over the past four decades of the HIV epidemic, researchers, clinicians and community organizations have learned to be creative in response to new advances, challenges and changing realities. The advent of well‐tolerated, simple regimens for effective HIV treatment and prevention has led to the recognition that we now have the tools to control the HIV epidemic. However, how to best adapt them to local circumstances remains of critical importance, and requires creativity, cultural awareness and the sharing of best practices. The COVID‐19 pandemic is another good case in point. Prior to March 2020, the use of telemedicine was a niche practice, and has since been increasingly utilized in the care of people living with HIV, as well as to assist individuals with counselling after self‐testing and to facilitate pre‐exposure prophylaxis initiation and monitoring.

Since its creation in 2004, JIAS—the *Journal of the International AIDS Society* has welcomed submission of papers that advance knowledge in implementation and science. This discipline is vital to the global HIV response, and offers valuable insights on providing, monitoring and enabling comprehensive, affordable and sustainable context‐specific treatment, prevention and care programmes. To stimulate further sharing of new insights garnered from creative responses to local epidemic challenges, JIAS is pleased to announce the inauguration of a new special feature for the journal: *Field Notes*. These are brief, focused articles that will describe the experiences of individuals working at the front lines of care, prevention, programme implementation and community engagement. They will define a specific concern—such as responses to providing optimal HIV care to the ways in which task‐shifting can augment service delivery—and discuss how a team assessed the problem and developed a unique solution that could be of interest to the broader AIDS community. *Field Notes* are intended to provide timely insights regarding contemporary challenges in HIV public health practice, including observations about the mechanisms of change, contextual influences and findings that emerge from implementation of evidence‐based interventions and programmes.

To launch this feature, we are pleased to present two articles that were coordinated and led by our *Field Notes* section Editors Drs. Nittaya Phanuphak [1] and Sunil Solomon [2]. These pieces reflect initiatives in Thailand and India that exemplify the types of implementation efforts we intend to share in the future.

The HIV epidemic has taught us many things that have informed other practices. Examples include the advent of ACT‐UP in the United States, which influenced the development of other advocacy movements, such as the Treatment Action Campaign in South Africa, and the use of nucleic amplification technology to quantify the response of HIV to antiretroviral therapy, which established a paradigm for monitoring other viral infections like hepatitis C. It is our hope that this new *Field Notes* section can continue to stimulate discussion while providing useful insights, disseminating best practices and promoting innovative ideas, with the ultimate goal of improving our service delivery for all people living with and affected by the HIV epidemic.

## COMPETING INTERESTS

AHS receives research and advocacy funding to her institution from ViiV Healthcare and Gilead Sciences. KHM has received unrestricted research grants from Gilead Sciences and Merck, Inc, and has served on scientific advisory boards for Gilead Sciences, Merck, Inc and ViiV Healthcare.

## AUTHORS’ CONTRIBUTIONS

KHM drafted the initial manuscript. AHS and MB contributed to critical review and revision of the paper. All authors have read and approved the final manuscript.

## FUNDING

None.

## DISCLAIMER

None.
